# Rapid antiretroviral initiation among Thai youth living with HIV in the National AIDS programme in the era of treatment at any CD4 cell count: a national registry database study

**DOI:** 10.1002/jia2.25574

**Published:** 2020-08-31

**Authors:** Sirinya Teeraananchai, Stephen J Kerr, Panthep Khananuraksa, Kiat Ruxrungtham, Thanyawee Puthanakit

**Affiliations:** ^1^ HIV‐NAT Thai Red Cross AIDS Research Centre Bangkok Thailand; ^2^ Department of Statistics Faculty of Science Kasetsart University Bangkok Thailand; ^3^ Biostatistics Excellence Centre Faculty of Medicine Chulalongkorn University Bangkok Thailand; ^4^ Kirby Institute University of New South Wales Sydney Australia; ^5^ National Health Security Office Bangkok Thailand; ^6^ Department of Medicine Faculty of Medicine Chulalongkorn University Bangkok Thailand; ^7^ Division of Infectious Diseases Department of Pediatrics Faculty of Medicine Chulalongkorn University Bangkok Thailand; ^8^ Center of Excellence in Pediatric Infectious Diseases and Vaccines Chulalongkorn University Bangkok Thailand

**Keywords:** youth living with HIV, antiretroviral therapy, universal coverage health programme, rapid ART, HIV cascade, loss to follow‐up

## Abstract

**Introduction:**

The process indicators of ending the HIV epidemic include 90% of people living with HIV receiving antiretroviral therapy (ART). The population of youth, however, has less access to healthcare. We assessed ART initiation and attrition outcomes of the HIV continuum from HIV diagnosis to ART initiation in youth living with HIV (YLHIV) and factors associated with ART initiation.

**Methods:**

We studied YLHIV aged 15 to 24 years who were registered on the National AIDS Program (NAP) from January 2008 to May 2019. The study period was divided into 2008 to 2013 (initiated ART by CD4‐guided criteria) and 2014 to 2018 (initiate ART at any CD4). Date of registration was used as a surrogate for the diagnosis date and defined as the baseline. The database included ART prescription and laboratory results, and the vital status was linked daily with the National Death Registry. Competing risk methods were used to assess factors associated with accessing ART, with loss to follow‐up (LTFU) and death considered as competing events. Logistic regression was used to assess factors associated with rapid ART initiation, defined as initiation ≤1 month after registration.

**Results:**

Overall, 51,607 youth registered on the NAP (42% between 2008 and 2013). Median age was 21 (IQR 20 to 23) years; 64% were male. Overall ART initiation was 80% in the first period and 83% in the second. The ART initiation rate was higher among YLHIV aged 15 to 19 years (86%) than 20 to 24 years (82%) (*p* < 0.001) in the second period. The proportion of youth starting rapid ART increased significantly from 27% to 52% between the two periods (*p* < 0.001). Factors associated with ART initiation were age 15 to 19 years (aSHR 1.09, 95% CI 1.06 to 1.11), female (aSHR 1.26, 95% CI 1.23 to 1.29) and registration year 2014 to 2018 (aSHR 1.73, 95% CI 1.69 to 1.76). The cumulative incidence of LTFU/death prior to ART initiation at 12 months was 3.8% (95% CI 3.6% to 4.1%) in the first period and 1.9% (95% CI 1.8% to 2.1%) in the second period.

**Conclusions:**

In the era of universal treatment of all at any CD4 level, 83% of YLHIV registered on the Thai National AIDS Program initiated ART. The majority initiated within one month of registration.

## 1 INTRODUCTION

Treating 90% of people who know they are living with HIV by 2030, the second step in the HIV care cascade, is an important UNAIDS 90:90:90 target [1]. Studies indicate that loss to follow up (LTFU) is high before people start antiretroviral therapy (ART) and more likely in these pre‐ART patients with high CD4 counts and less advanced clinical stage [2, 3, 4], due to waiting times for ART eligibility [5]. Youth living with HIV (YLHIV) have traditionally had poor outcomes, with high LTFU and death rates before ART initiation, and even lower proportions achieve the final endpoint of viral suppression in the cascade of care [6, 7, 8]. Evidence from Africa and Thailand suggests that the rate of LTFU before ART initiation in young people (age 15 to 24 years) is higher than the rates observed in adults (age ≥25 years) and early adolescents (age <15 years) [3, 9, 10].

In Thailand, people living with HIV have been offered free HIV care and treatment through the universal coverage National AIDS Program (NAP) from the end of 2007 [11]. Since then, there have been successive updates in the CD4 thresholds set for starting ART (CD4 < 200 cell/mm^3^ from 2008 to 2010, CD4 < 350 cells/mm^3^ from 2011 to 2013, and any CD4 level since 2014) [12]. The updated HIV treatment guidelines have led to overall increases in the number of patients accessing ART: in 2017, 87% of all people living with HIV registered on the NAP accessed ART and 69% were virally suppressed [13].

However, although the largest group of new HIV diagnoses is in young people who acquired HIV sexually, particularly young men who have sex with men [14, 15, 16], no outcome data along the care cascade are available for this group.

We sought to assess and compare outcomes for YLHIV treated through the Thai NAP at the second step of the care cascade, for 2008 to 2013 and for 2014 to 2018 when guidelines changed to ART initiation recommended at any CD4 count. Our primary aim was to assess changes in ART initiation and attrition cumulative incidence after HIV diagnosis in YLHIV. Our secondary aims were to assess factors associated with ART initiation and ART initiation within 30 days of registration on the NAP.

## 2 METHODS

### 2.1 Study cohort from national registry database

Briefly, health insurance programmes in Thailand consist of the Social Security Scheme for people working in the private sector, the Civil Servant Medical Benefit Schemes for people working in the public sector and the Universal Coverage Scheme for those not covered by either of the other two schemes. For HIV management, all these insurance systems were integrated as the NAP and have been administered by the National Health Security Office (NHSO) since 2007.

The NAP database was established in 2007 as a registration and reimbursement portal for HIV programmes. Hospitals and clinical sites enter laboratory results, clinical information and ART dispensing records into the database system at registration (HIV diagnosis) and all subsequent visits. CD4 tests are provided every six months and HIV‐RNA is measured six months after starting ART and annually thereafter. ART is provided based on national HIV treatment guidelines [11]. Since site cost reimbursement is contingent on data being entered for services provided, the data are highly accurate. In addition, the database is linked with the national death registry, so patient vital status is updated daily.

More than 95% of YLHIV are covered by the Universal Coverage Scheme. An effective prevention of mother‐to‐child transmission (PMTCT) programme has seen vertical transmission decline to <1% of live births [17], but young people who acquired HIV sexually now account for the biggest number of new infections [18, 19]. Accordingly, the concept of treating YLHIV has become more flexible, allowing YLHIV to start ART without parental consent, consistent with treating at any CD4 level. Moreover, YLHIV are cared for in paediatric clinics if they are younger than 18 years and then mostly transferred to an adult clinic at the age of 18 years.

This study was approved by the Institutional Review Board of the Institute for Development of Human Research Protection, Ministry of Public Health, Thailand. A waiver of consent was granted for this data analysis. The NAP database was de‐identified by the NHSO before analysis.

### 2.2 Study population

This study included YLHIV aged 15 to 24 years who registered on the NAP from January 2008 to May 2018. Follow‐up data were available until August 2019, providing at least a year of follow‐up for all patients to assess outcomes, including an additional three months to account for possible delays in data entry. We excluded patients who were treated before enrolment into the NAP since no clinical information was available at the time of HIV diagnosis or ART initiation.

### 2.3 Definitions and outcomes

Date of registration was used as a surrogate for diagnosis date and defined as the baseline.

The primary outcome was ART initiation, defined as patients starting ART after registration throughout the study period. Secondary outcomes were as follows:

1. Rapid ART initiation, defined as starting ART on the same day, within seven days and within one month of registration
2. Attrition outcomes (LTFU, mortality) from HIV diagnosis to ART initiation prior to ART initiation.

The study endpoints were ART initiation after HIV diagnosis, LTFU and death; those who did not start ART were censored at their last clinic visit. LTFU was defined as not starting ART and not attending clinic for 12 months after their previous appointment unless they subsequently returned to care before the final data transfer date. Patients who died without starting ART were classified as dead even if the patient was previously lost to follow‐up. Patients who did not reach a study endpoint (starting ART, LTFU or death) were censored at the date of their most recent clinic visit. Baseline CD4 was taken as the closest result available to the date of registration within a window of six months before or after registration. Pre‐ART CD4 results were defined as the closest results within a window 12 months before and up to two months after the date of ART initiation. Vital status was ascertained by linkage with death registry, which was updated daily.

### 2.4 Statistical analysis

Baseline characteristics, including demographics, age (15 to 19 years were classified as older adolescents and 20 to 24 years as young adults [20]), year, region, history of opportunistic infection [20] and CD4 count level, were summarized using descriptive statistics by period of registration. The first period was defined as 2008 to 2013 and the second as 2014 to 2018. The outcomes along the HIV cascade from HIV diagnosis to ART initiation were reported as proportions by period of registration. The competing risk method of Fine and Gray [21] was used to calculate subdistribution hazard ratios (SHR), to assess associations between baseline characteristics and ART initiation, with LTFU and death considered as competing events.

From these analyses, the cumulative incidence of ART initiation and of LTFU/death was generated using competing risk estimators. Logistic regression was used to assess predictors of rapid ART initiation (within one month of registration). In this analysis, those starting ART within one month of registration were considered as successes and those who did not start within one month for any reason, including LTFU or death, were considered as failures. Covariates assessed in both logistic and competing risks models included baseline age, gender, country region, year of registration and history of opportunistic infections.

Variables with *p* < 0.10 were included in multivariate models. Statistical significance was identified using a two‐sided *p* < 0.05. Statistical analysis was performed with SAS version 9.4 (SAS Institute Inc, Cary, NC, USA) and with Stata version 14 (Statacorp, College Station, TX, UAS).

## 3 RESULTS

A total of 52,397 youth registered on the NAP from 2008 to 2018; 790 youth (2%) were excluded because they initiated ART in other facilities, such as private clinics and research programmes. Therefore 51,607 newly diagnosed patients were analysed, of whom 21,825 (42%) registered from 2008 to 2013 and 29,782 (58%) from 2014 to 2018.

### 3.1 Patient characteristics

Baseline characteristics by period of registration are shown in Table 1. The median age was 21 (interquartile range [IQR] 20 to 23) years. Most patients (75%) enrolled at age 20 to 24 years versus age 15 to 19 years. The proportion of males increased from 50% to 74% from the first to the second period. A quarter of the YLHIV were from the northeast and the next highest proportions were from the northern and Bangkok regions. Fifty‐one percent of YLHIV had CD4 counts available at registration and the median CD4 was 333 (IQR 171 to 491) cells/mm^3^ Only 48% of YLHIV who registered had pre‐ART CD4 available: the median pre‐ART CD4 count was 255 (IQR 81 to 395) cells/mm^3^ in the first period and 318 (IQR 163 to 468) cells/mm^3^ in the second period.

**Table 1:** Baseline characteristics of youth living with HIV aged 15 to 24 years registered on the Thai National AIDS Program in 2008 to 2018

Baseline characteristics^a^	2008 to 2013	2014 to 2018	Total	*p* ^b^
N	21,825	29,782	51,607	
Median age at registration, years	22 (19 to 23)	21 (20 to 23)	21 (20 to 23)	<0.001
Age group				
15 to 19 years	5546 (25)	7333 (25)	12,879 (25)	<0.001
20 to 24 years	16,279 (75)	22,449 (75)	38,728 (75)	
Male	11,013 (50)	22,075 (74)	33,088 (64)	<0.001
History of opportunistic infection				
Yes	2105 (10)	1588 (5)	3693 (7)	<0.001
No	19,720 (90)	28,194 (95)	47,914 (93)	
CD4 available at registration	4682 (21)	21,612 (73)	26,294 (51)	
Median (IQR) CD4 at registration, cells/mm^3^	331 (156 to 492)	333 (175 to 491)	333 (171 to 491)	0.22
Pre‐ART CD4 available	6626 (30)	17,900 (60)	24,526 (48)	
Median pre‐ART CD4 count (cells/mm^3^)	255 (81 to 395)	318 (163 to 468)	300 (137 to 451)	<0.001
Geographical regions				
Bangkok metropolitan	2987 (14)	5609 (19)	8596 (17)	<0.001
Northeast	6015 (28)	7651 (26)	13,666 (26)	
North	3853 (18)	5079 (17)	8932 (17)	
Central	3397 (16)	5092 (17)	8489 (16)	
East	2225 (10)	2900 (10)	5125 (10)	
South	2440 (11)	2265 (8)	4705 (9)	
West	908 (4)	1186 (4)	2094 (4)	

ART, antiretroviral therapy.

^a^ Presented as n (%) for categorical data and median (interquartile range) for continuous data

^b^ The comparisons were performed using Pearson’s Chi‐square tests for categorical data, and a Wilcoxon rank sum test for continuous data.

### 3.2 Study outcomes

**Figure 1:**
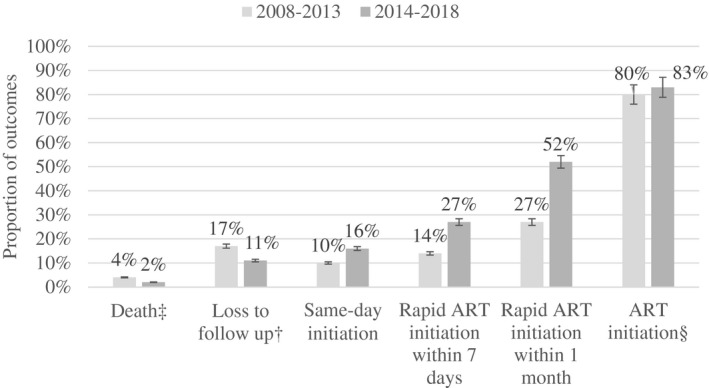
Outcomes of youth living with HIV aged 15 to 24 years after registered on the Thai National AIDS Program. ^§^ART initiation was defined as patients starting ART after registration throughout study period. ^†^LTFU was defined as not starting ART and not attending clinic for 12 months after their previous appointment, unless they subsequently returned to care before the final data transfer date. ^‡^Patients who died without starting ART were classified as dead even if they were previously loss to follow‐up. There were statistically significant differences in the percent of youth experiencing all outcomes in 2008 to 2013 compared with 2014 to 2018 (*p* < 0.001). Formal comparisons were made using Pearson’s Chi‐square. Error bars represented 95% confidence interval. ART, antiretroviral therapy; LTFU, loss to follow up

The percentage of YLHIV achieving outcomes along the HIV cascade from HIV diagnosis to ART initiation, by study period, is shown in Figure 1. The percentage initiating ART throughout the study increased from 80% to 83% from 2008‐2013 to 2014‐2018 (*p* < 0.001). We found statistically significant improvements in the percentage of youth initiating ART on the same day, within seven days and within one month of registration in the first versus the second period. The percentage of deaths before ART initiation in the first period decreased from 4% to 2% in the second period (*p* < 0.001). In the recent period where guidelines advocated starting ART at any CD4 count, the percentage of patients who were lost to follow‐up prior to ART initiation reduced from 17% to 11% (*p* < 0.001).

Moreover, the overall median duration from registration to ART initiation was 1.47 (IQR 0.37 to 12.18) months, with a total of 42,914 person‐years. The median time from registration to ART initiation in 2008 to 2013 was six (IQR 0.8 to 25) months, significantly longer than in the second period [one (IQR 0.2 to 4) month, *p* < 0.001]. The crude mortality rate was 3.52 (95% CI 3.35 to 3.70) per 100 person‐years. The crude LTFU rate was 16.11 (95% CI 15.74 to 6.50) per 100 person‐years.

**Figure 2:**
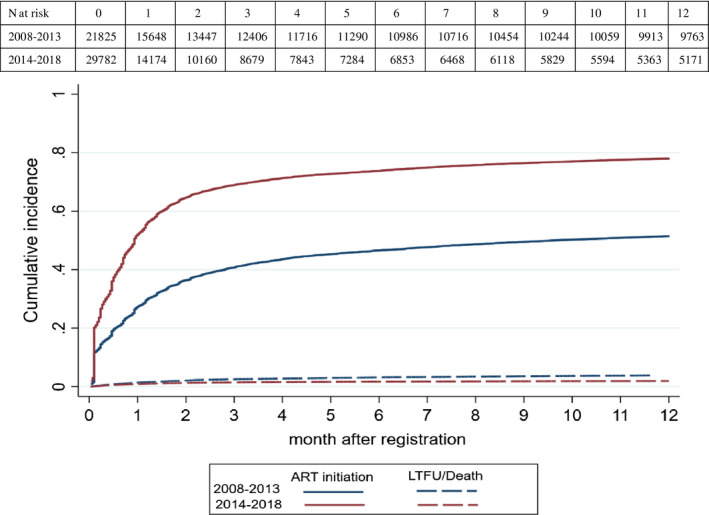
Cumulative incidence of accessing antiretroviral therapy among youth living with HIV on the Thai National AIDS Program, by period of registration. The cumulative incidence was calculated by competing risk models. ART, antiretroviral therapy; LTFU, loss to follow up.

The cumulative incidence of initiating ART for those who registered from 2008 to 2013 at one, three, six and 12 months was 26.9% (95% CI 26.3% to 27.5%), 40.6% (95% CI 40.0% to 41.3%), 46.5% (95% CI 45.9% to 47.2%) and 51.4% (95% CI 50.8% to 52.1%) respectively. Among those who registered from 2014 to 2018, the cumulative incidence of ART initiation at one, three, six and 12 months was 51.5% (95% CI 51.0% to 52.1%), 68.8% (95% CI 68.3% to 69.4%), 73.8% (95% CI 73.3% to 74.3%) and 78.0% (95% CI 77.5% to 78.4%) respectively. The cumulative incidence of LTFU/death prior to ART initiation at 12 months was 3.8% (95% CI 3.6% to 4.1%) in the first period and 1.9% (95% CI 1.8% to 2.1%) in the second period (Figure 2).

### 3.3 Factors associated with antiretroviral treatment initiation

We assessed factors associated with ART initiation after NAP registration. All covariates from univariate analysis were included in the multivariate analysis (Table 2). Patients aged 15 to 19 years who registered had a 9% higher chance of initiating ART compared with those aged 20 to 24 years who enrolled (aSHR 1.09, 95% CI 1.06 to 1.11). Females (aSHR 1.26, 95% CI 1.23 to 1.29) were more likely to initiate ART than males. Young people with no history of opportunistic infection were less likely to access ART (aSHR 0.95, 95% CI 0.91 to 0.98). In addition, youth who registered from 2014 had a higher chance (aSHR 1.73, 95% CI 1.69 to 1.76) of initiating ART versus those who registered before 2014. Patients living in the southern, eastern and western regions of Thailand had a lower chance of starting ART than those who lived in Bangkok Metropolitan area.

**Table 2:** Factors associated with accessing antiretroviral treatment from a competing risks regression model

Characteristics (n = 51,607)^a^	Univariate	*p*	Multivariate	*p*
	SHR (95% CI)		aSHR (95% CI)	
Age group		<0.001		<0.001
15 to 19 years	1.10 (1.08 to 1.13)		1.09 (1.06 to 1.11)	
20 to 24 years	Reference		Reference	
Sex		<0.001		<0.001
Male	Reference		Reference	
Female	1.09 (1.06 to 1.11)		1.26 (1.23 to 1.29)	
History of opportunistic infection		0.04		0.001
Yes	Reference		Reference	
No	1.03 (1.01 to 1.07)		0.95 (0.91 to 0.98)	
Year of registration		<0.001		<0.001
2008 to 2013	Reference		Reference	
2014 to 2018	1.62 (1.59 to 1.66)		1.73 (1.69 to 1.76)	
Geographical region		<0.001		<0.001
Bangkok Metropolitan	Reference		Reference	
Northeastern	0.94 (0.91 to 0.97)		0.98 (0.94 to 1.01)	
Northern	0.93 (0.90 to 0.96)		0.97 (0.94 to 1.00)	
Central	0.95 (0.92 to 0.99)		0.97 (0.93 to 1.00)	
Southern	0.85 (0.82 to 0.88)		0.90 (0.86 to 0.93)	
Eastern	0.88 (0.85 to 0.92)		0.89 (0.86 to 0.93)	
Western	0.76 (0.72 to 0.80)		0.76 (0.72 to 0.81)	

aSHR, adjusted subdistribution hazard ratio; SHR, subdistribution hazard ratio.

^a^ Characteristics were evaluated at baseline.

### 3.4 Factors associated with rapid antiretroviral treatment initiation

In logistic regression analyses assessing starting ART within one month of registration (Table 3), all covariates tested in univariate models were included in the multivariate analysis. Patients aged 15 to 19 years who registered (aOR 1.14, 95% CI 1.10 to 1.19) were more likely to start ART within one month than those aged 20 to 24 years who enrolled. Females had 61% (aOR 1.61, 95% CI 1.54 to 1.68) higher odds of rapidly starting ART than males. Patients with no history of opportunistic infection were more likely to start ART within one month than those with history. The odds of starting ART rapidly increased threefold (aOR 3.20, 95% CI 3.07 to 3.33) in patients who registered in the period 2014 to 2018 versus those registered in 2008 to 2013. Patients living in the Bangkok Metropolitan area had higher odds of rapidly starting ART than other country regions.

**Table 3:** Factors associated with rapid antiretroviral treatment initiation within one month after registration in the Thai National AIDS Program

Characteristics^a^ (n = 51,607)	Starting ART within one month, n (%)	Univariate^b^	*p*	Multivariate^b^	*p*
		OR (95% CI)		aOR (95% CI)	
Age group			<0.001		<0.001
15 to 19 years	5795 (27)	1.20 (1.15 to 1.25)		1.14 (1.10 to 1.19)	
20 to 24 years	15,684 (73)	Reference		Reference	
Sex			<0.001		<0.001
Male	13,330 (62)	Reference		Reference	
Female	8149 (38)	1.16 (1.12 to 1.21)		1.61 (1.54 to 1.68)	
History of opportunistic infection			<0.001		<0.001
Yes	1092 (5)	Reference		Reference	
No	20,387 (95)	1.76 (1.64 to 1.90)		1.43 (1.33 to 1.54)	
Year of registration			<0.001		<0.001
2008 to 2013	5971 (28)	Reference		Reference	
2014 to 2018	15,508 (72)	2.88 (2.78 to 2.99)		3.20 (3.07 to 3.33)	
Geographical regions			<0.001		<0.001
Bangkok Metropolitan	4120 (17)	Reference		Reference	
Northeastern	5690 (26)	0.78 (0.73 to 0.82)		0.80 (0.76 to 0.85)	
Northern	3730 (17)	0.78 (0.73 to 0.83)		0.81 (0.76 to 0.86)	
Central	3555 (16)	0.78 (0.74 to 0.83)		0.76 (0.71 to 0.81)	
Eastern	2026 (10)	0.71 (0.66 to 0.76)		0.70 (0.65 to 0.75)	
Southern	1712 (9)	0.62 (0.58 to 0.67)		0.67 (0.62 to 0.72)	
Western	646 (4)	0.48 (0.44 to 0.54)		0.46 (0.42 to 0.51)	

aOR, adjusted odd ratio; OR, odd ratio.

^a^ Characteristics were evaluated at baseline

^b^ The analyses were performed using Logistic regression models.

## 4 DISCUSSION

This study using the national registry database of the Thai National AIDS Program is the first to specifically address outcomes along the care cascade from HIV diagnosis to antiretroviral therapy initiation among young people aged 15 to 24 years. It reflects real‐life practice and outcomes in a low‐ to middle‐income country setting, where universal coverage is provided, in response to changes in national treatment guidelines. Our study showed that ≥80% of YLHIV initiated ART in both periods. The proportion initiating ART within one month of registration increased from 27% to 52%, and within six months of registration, it increased from 47% to 74%, coinciding with guidelines changing to starting ART at any CD4 count [22]. This finding likely reflects the effect of the guideline change, in association with more experience in treatment sites and better infrastructure over the years.

Our study shows an increase in the trend of rapidly initiating ART in YLHIV during the study period. From 2014, more than 50% started ART within one month, increasing to 69% at three months and 78% at 12 months. This is higher than found by the International Epidemiology Databases to Evaluate AIDS (IeDEA) Global Cohort in young people aged 15 to 19 years (37.4% at one month, 62.2% at 12 months) [23]. Moreover, the median time from HIV diagnosis to ART initiation in our study decreased from six to one month after guidelines recommended starting ART irrespective of CD4 count levels. This finding is similar to a study from Zambia and South Africa in the period 2004 to 2015, where median time reduced after delivering an intervention to offer ART regardless of CD4 count (the HPTN 071, PopART, trial) from ten to six months [24].

The increase in cumulative incidence of ART initiation in YLHIV provides evidence of the changes in response to updates in the national HIV treatment guidelines. This finding is consistent with a meta‐analysis that found the largest changes in cumulative incidence of ART initiation were in those who were ineligible under prior guidelines and in patients aged 16 to 24 years [25].

Our analysis of predictive factors for initiating ART and rapid ART showed that rates were especially high in those who were younger (15 to 19 years) and in females. This is similar to findings from an African study, where those who initiated same‐day ART were younger, more likely to be female and presented with less advanced clinical disease [26, 27]. With Thailand’s PMTCT programme, all pregnant women are tested for HIV during pregnancy, so most female patients confirmed as HIV positive during pregnancy have prompt ART initiation; previous studies support evidence that rapid ART initiation in pregnancy is effective in reducing mother to child transmission [3, 27, 28, 29].

Furthermore, patients who lived in the Bangkok area were more likely to access ART than those in the southern, western and eastern regions. One possible explanation is that the central (including Bangkok), northeast and northern regions have the highest prevalence of HIV and were the earliest regions affected by the Thai HIV epidemic. They therefore have more experience in managing HIV patients and perhaps have more specialized HIV clinics. Previous studies from Zimbabwe and China support evidence that patients who live in urban areas can more easily access ART or treatment than those who live in rural areas [30, 31].

While our findings are encouraging, they also show that further and more aggressive efforts are needed to achieve the second and third goals of the treatment cascade: 90% of people diagnosed with HIV receiving ART; and 90% of these ART‐treated YLHIV virally suppressed. Integration of multiple effective approaches from policy, hospital services, healthcare centre services and community‐led services are essential strategies to reach this ultimate goal.

Previous studies indicate that attrition rates are high during the period from HIV testing to ART initiation when eligibility criteria are based on CD4 thresholds, particularly in YLHIV [2, 3, 5, 7, 23, 32]. Since guidelines have recommended immediately starting ART in all people living with HIV, high rates of patient attrition in the period between HIV diagnosis and ART initiation have reduced [2, 33, 34]. LTFU before ART initiation in our study reduced from 17% to 11% in the period of recommending treatment at any CD4 count.

Meanwhile, the IeDEA Global Cohort, which is comprised of cohorts with different infrastructures, reported that overall LTFU (defined as clinic absence of >6 months) before starting ART was 20.4%, but only 2.3% in the Asia‐Pacific region [35]. The majority of Asian cohorts in the IeDEA study were from research centres that specifically cater for YLHIV. These cohorts might therefore have a selection bias for better outcomes compared with the national registry database and with other world regions where LTFU is high due to undocumented mortality. However, similarly encouraging evidence was found by a Rwandan study where mortality and LTFU before ART initiation reduced after the change in Rwanda’s national ART eligibility guidelines [36]. Offering ART immediately at diagnosis improves retention in care and mitigates LTFU in patients who are not ready to start ART [37].

There are a number of limitations to this study. First, we defined the date of registration with NAP as the surrogate for date of HIV diagnosis, which could create bias by shortening the perceived time from HIV diagnosis to ART initiation, as some patients may have been diagnosed at HIV testing centres or private clinics and re‐tested at NAP registration. However, this seems unlikely because testing within the NAP is free and only 2% of NAP patients were known to be diagnosed outside the programme, indicating that our results are applicable to the vast majority of Thai youth diagnosed with HIV.

Second, the rate of initiating ART in the NAP may be lower than observed in our study since a small proportion of patients may access ART through research programmes and private clinics. We did not have detailed information about service delivery, whether hospitals had specialized HIV clinics, or hospital size; we had information only on location of hospital. These factors might impact on ART access in young people living outside Bangkok. For this reason, we could not definitively establish whether these structural factors influenced the differences we noted between urban and rural areas. Third, we were unable to link PMTCT programme information with the NAP data, so we cannot present separate data about females who were diagnosed during pregnancy in the study period. We also did not have data on mode of infection in the database. We assume that the majority of YLHIV diagnosed with HIV aged 15 to 24 years acquired HIV through sexual transmission.

Fourth, approximately 50% of baseline CD4 counts were not available in this study. Fifth, the NHSO database does not record details about socio‐economic factors, such as caregiver status, education status, occupation and income, so we were precluded from examining the effects of these factors which might also have an impact on access to ART.

Last, this is an observational study and it is possible that the outcomes in our study may be subject to unobservable biases and confounding. Regression discontinuity or interrupted time series analysis would allow exploration of temporal trends that may have already been underway in Thailand before the guidelines recommended treatment at any CD4 count.

Nevertheless, we believe that our method is also valid to assess changes related to treatment guidelines since no improvement trends were evident by calendar time prior to 2014. In addition, our model was able to assess the cumulative incidence of ART initiation and attrition simultaneously in the same mode. Furthermore, the changes observed from our first to second study period coincided with a structural change in the National Treatment Guidelines on HIV/AIDS Treatment, affecting the entire country in a period with stable availability of antiretroviral agents, which lends support to the validity of our findings.

## 5 CONCLUSIONS

In the period 2014 to 2018 when Thai guidelines advocated initiation of ART at any CD4 count, 83% of young people initiated ART and more than half of YLHIV initiated ART within one month of registration. This more rapid initiation also likely influenced the lower rates of mortality and LTFU prior to ART initiation seen in this period. Continued improvement in early access to ART is needed to reach the target of treating 90% of YLHIV with ART. Moreover, initiating ART rapidly can help control the HIV epidemic and optimize the health of people living with HIV.

## COMPETING INTERESTS

KR received the Senior Research Scholarship from the Thailand Research Fund (TRF). He also received honoraria or consultation fees from Merck, Roche, Jensen‐Cilag, Tibotec, Mylan and the Governmental Pharmaceutical Organization (GPO, Thailand). He has participated in a company‐sponsored speaker’s bureau from Abbott, Gilead, Bristol‐Myers Squibb, Merck, Roche, Jensen‐Cilag, GlaxoSmithKline and GPO. TP received a scholarship from the Anandamahidol Foundation and clinical research grant from ViiV. ST was funded as a CIPHER grantee from the International AIDS Society in 2018 to 2020.

## AUTHORS’ CONTRIBUTIONS

ST, SK, KR and TP created the study concept and study design. ST, SK, PK and KR were responsible for data collection or oversaw programme implementation. ST conducted the analysis. SK, KR and TP advised on the analysis. ST, SK, PK, KR and TP interpreted the data. ST drafted the manuscript. All authors critically reviewed the manuscript and approved the manuscript for submission.

## ABBREVIATIONS

NAP, National AIDS Program; LTFU, loss to follow up; ART, antiretroviral therapy; aSHR, adjusted subdistribution hazard ratio; aOR, adjusted odds ratio; IeDEA, the International Epidemiology Databases to Evaluate AIDS; IQR, interquartile range; PYFU, patient‐years of follow‐up; 95% CI, 95% confidence interval; PMTCT, prevention of mother‐to‐child transmission.
